# Plasma sCD14 as a Biomarker to Predict Pulmonary Exacerbations in Cystic Fibrosis

**DOI:** 10.1371/journal.pone.0089341

**Published:** 2014-02-20

**Authors:** Bradley S. Quon, David A. Ngan, Pearce G. Wilcox, S. F. Paul Man, Don D. Sin

**Affiliations:** 1 Centre for Heart Lung Innovation, St. Paul’s Hospital, University of British Columbia, Vancouver, British Columbia, Canada; 2 Division of Respiratory Medicine, St. Paul’s Hospital, University of British Columbia, Vancouver, British Columbia, Canada; University of Tübingen, Germany

## Abstract

**Background:**

One in four cystic fibrosis (CF) patients diagnosed with a pulmonary exacerbation will not recover their baseline lung function despite standard treatment. This highlights the importance of preventing such events. Clinical decision-making can be improved through a simple blood test that predicts individuals at elevated short-term risk of an exacerbation.

**Methods:**

We obtained plasma samples from 30 stable CF patients from the St. Paul’s Hospital Adult CF Clinic (Vancouver, Canada). For 15 patients, an additional plasma sample was obtained during an exacerbation. Soluble CD14 (sCD14) and C-reactive protein (CRP) were quantified using ELISA kits. Myeloperoxidase (MPO), interleukin(IL)-6, IL-1β, monocyte chemoattractant protein-1 (MCP-1), vascular endothelial growth factor (VEGF), and granulocyte colony-stimulating factor (G-CSF) were quantified using Luminex™ immunoassays. Stable state biomarker levels were examined in their ability to predict individuals that would experience a pulmonary exacerbation requiring intravenous (IV) antibiotics within 4 months. Paired stable and exacerbation plasma biomarker levels were also compared.

**Results:**

sCD14 levels were significantly higher in patients that experienced a pulmonary exacerbation requiring IV antibiotics within 4 months (p = 0.001). sCD14 cut-off value of 1450 ng/mL was associated with an area under the curve of 0.91 (95% CI 0.83–0.99) for predicting an exacerbation within 4 months of a stable visit, with a sensitivity of 100% and specificity of 82%. Plasma sCD14 levels were significantly higher during exacerbations than during periods of clinical stability (p = 0.03).

**Conclusions:**

Plasma sCD14 is a promising biomarker for identifying CF patients who will exacerbate within 4 months of a stable visit but requires further study in larger, independent cohorts.

## Introduction

Cystic fibrosis (CF) pulmonary exacerbations are clinically important events for CF patients. They are associated with reduced quality of life and survival [1], [2], [3], [4]. Despite aggressive treatment with intravenous (IV) antibiotics targeting airway infection, one in four CF patients diagnosed with an exacerbation will fail to regain their prior baseline lung function post-treatment [5]. This suggests exacerbations may lead to irreversible lung damage, thus highlighting the importance of exacerbation prevention and early diagnosis.

Although CF pulmonary exacerbation definitions vary, they are characterized by a combination of changes in symptoms and lung function (e.g. FEV1% predicted) necessitating an augmentation in treatment [6]. However, because these changes occur downstream from the underlying pathogenic processes, there is invariably a delay of several days (if not weeks) between onset of the exacerbation and diagnosis and treatment of these events. This delay contributes to incomplete recovery of patients’ health status and lung function. To prevent these delays, however, clinicians would require a highly sensitive and specific (and inexpensive) test that can accurately risk-stratify these patients for imminent exacerbation even when their symptoms and lung function are stable. Exacerbation outcomes can potentially be improved by identifying patients that are at elevated short-term risk of developing an exacerbation. Such individuals can be selectively targeted for initiation or intensification of maintenance therapies to prevent exacerbations. Exacerbations can potentially be prevented with medications such as mucolytic agents and inhaled antibiotics [7]. These individuals can also be monitored more closely, which may lead to earlier exacerbation detection and treatment. Biomarkers reflective of systemic inflammation have demonstrated promise in monitoring CF lung disease activity, even when patient symptoms are stable [8]. Systemic inflammatory changes likely occur upstream from changes in clinical parameters and thus may enhance exacerbation risk-stratification.

The objectives of this study were to identify systemic inflammatory biomarkers that can be used to: 1) predict CF individuals that are at elevated risk of developing an exacerbation within 4 months of a stable visit; and 2) discriminate between CF individuals that are clinically stable vs. those experiencing an exacerbation.

## Methods

### Study Population

We enrolled 64 adult patients from the Adult Cystic Fibrosis (CF) Clinic at St. Paul’s Hospital (Vancouver, British Columbia, Canada) between April 2009 and June 2010 who were clinically stable at the time of baseline assessment. We included patients with two disease-causing CF transmembrane conductance regulator (CFTR) mutations and at least two sweat chloride measurements of greater than 60 mmol/L. Patients who had experienced an exacerbation within 4 weeks of the initial stable visit or had previously undergone a solid organ transplant were excluded. Patients were not required to have a prior history of exacerbations. Written informed consent was obtained from the research subjects and the University of British Columbia – Providence Health Care Research Ethics Board (UBC-PHC REB) approved the study protocol and consent procedure.

### Blood Collection

Venous blood samples were collected in clinic, processed for plasma, and stored at −80°C until thawing for batched analysis. Blood was collected during the initial stable assessment and longitudinally during subsequent stable or exacerbation clinic visits up to August 2010. If an enrolled patient missed a clinic appointment, they were not approached outside of clinic to obtain blood samples. Blood samples were classified as being from a stable clinic visit if the subject did not have a significant change in symptoms warranting escalation in therapy within 4 weeks of the blood draw. Blood samples were classified as being from an exacerbation clinic visit if the subject had the need for additional antibiotic treatment (oral or intravenous) as indicated by a recent change in at least two of the following: change in sputum volume or colour, increased cough, increased malaise, fatigue or lethargy, anorexia or weight loss, increased dyspnea, decrease in pulmonary function by 10% or more, radiographic changes [6], [9]. Blood was not collected if the patient was already started on antibiotics prior to the exacerbation clinic visit. CF physicians were not blinded to study participation but were blinded to the blood biomarker results, which were performed several months following the clinic visit.

### Clinical Information

Spirometric data were obtained using standard techniques, in accordance with guidelines from the American Thoracic Society [10]. Demographic and other clinical data were extracted through chart review. Exacerbations during the follow-up period were ascertained through chart review (up to 2 years following enrollment) by study personnel blinded to the biomarker results. Because follow-up exacerbations were based on chart review, we could not reliably separate out the use of oral antibiotics for prophylaxis, or extra-pulmonary indications (e.g. sinusitis) versus those used for acute exacerbations. Thus, follow-up exacerbations were defined based on the need for intravenous antibiotic treatment as indicated by a recent change in at least two of the following: change in sputum volume or colour, increased cough, increased malaise, fatigue or lethargy, anorexia or weight loss, increased dyspnea, decrease in pulmonary function by 10% or more, radiographic changes [6], [9]. Infection status was defined based on the sputum culture results reported at the time of the CF clinic encounter.

### Samples Analyzed

**Figure 1:**
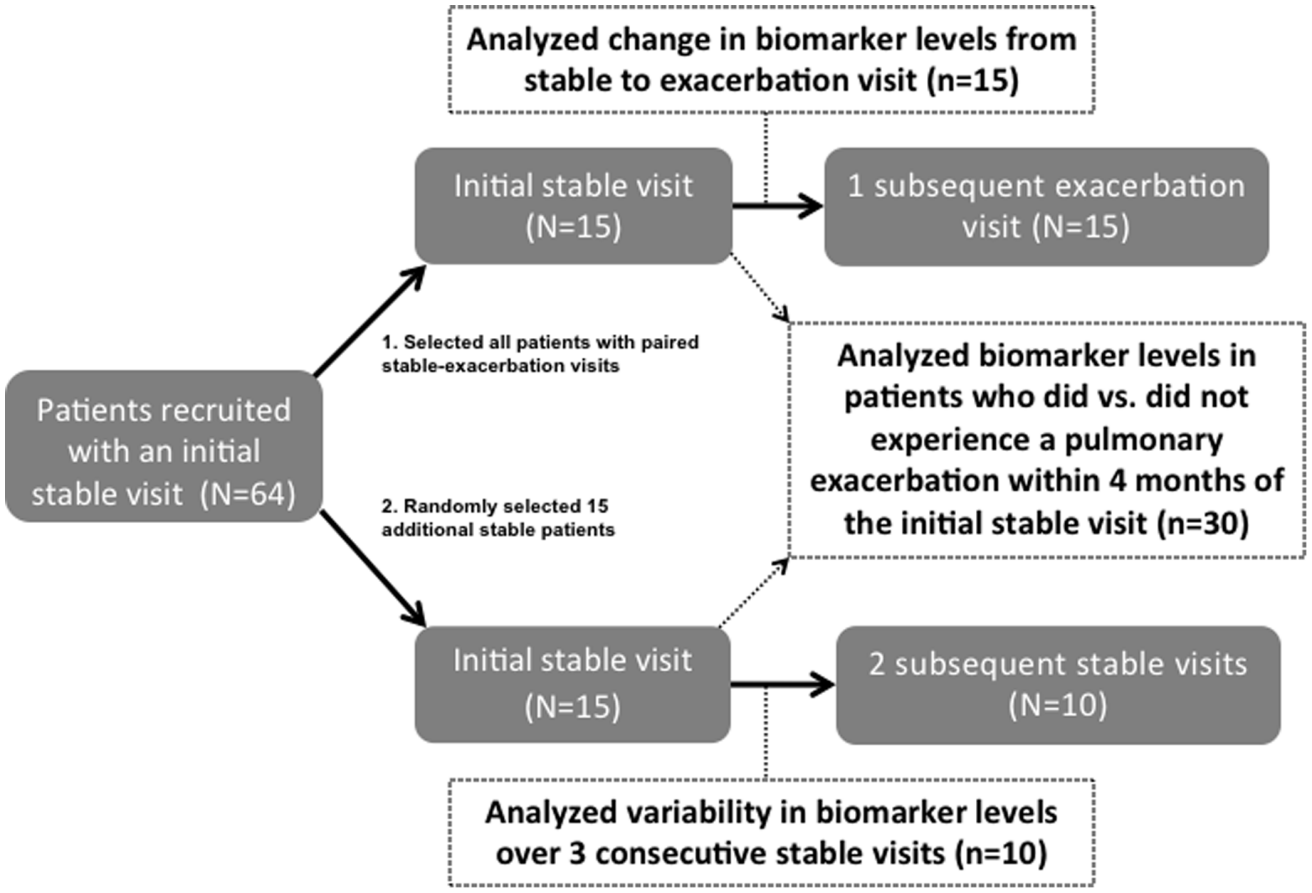
Blood collection schedule and analysis plan.

*A priori*, we decided to analyze plasma samples from 30 out of 64 patients in this pilot study. We selected all 15 (out of 64) patients who had a paired sample collected during clinical stability followed by an exacerbation visit. We then randomly selected an additional 15 patients from the remaining 49 patients. All 15 patients had at least one initial stable sample and 10 of the 15 patients had 2 subsequent stable samples to allow for the analysis of variability in measurements over 3 consecutive stable clinic visits (**Figure 1**).

### Biomarker Assays

Circulating proteins from the following inflammatory pathways were selected based on review of the literature and the availability of reliable commercial immunoassays: 1) acute phase proteins; 2) pro-inflammatory cytokines; 3) markers of neutrophilic inflammation; and 4) markers of tissue injury/hypoxia. Acute phase proteins included C-reactive protein (CRP) and soluble cluster of differentiation 14 (sCD14), which were measured using enzyme-linked immunosorbent assay (ELISA) kits (R&D Systems, Minneapolis, MN) with a coefficient of variation of 2.8% and 3.1%, respectively. Cytokines involved in the early phase inflammatory response included interleukin-6 (IL-6), IL-1β, and monocyte chemoattractant protein-1 (MCP-1), which were quantified using high-sensitivity Luminex**®** bead-based multiplex assays (R&D Systems, Minneapolis, MN) with a coefficient of variation of 3.5%, 6.9%, and 3.3%, respectively. Markers of neutrophilic inflammation included granulocyte colony-stimulating factor (G-CSF) and myeloperoxidase (MPO), and a marker of tissue injury/hypoxia included vascular endothelial growth factor (VEGF). These markers were also quantified using high-sensitivity Luminex**®** bead-based multiplex assays (R&D Systems, Minneapolis, MN) with a coefficient of variation of 5.3%, 3.9%, and 5.7% respectively. All samples were measured in duplicate and by study personnel who were blinded to patient characteristics and to the study visit number. To mitigate batch effect, baseline and exacerbation paired samples were assayed on the same plates.

### Statistical Analysis

Descriptive statistics were presented as medians/interquartile ranges or numbers/proportions. The relationship between stable visit biomarker level and FEV1% predicted was examined using Spearman’s rank correlation coefficient. Variability in biomarker measurement, including FEV1% predicted, over three consecutive stable clinic visits was examined using a coefficient of variation (calculated as standard deviation divided by the mean).

Following log transformation to normalize the data, we used the two-group t-test to compare stable visit biomarker levels in patients who did vs. did not develop a pulmonary exacerbation requiring IV antibiotics within 4 months. For biomarkers that were significantly different between the two groups, we performed an area under receiver operating characteristic (ROC) curve analysis to evaluate various biomarker cut-off values to predict exacerbations requiring IV antibiotics within 4 months of the stable visit. We also conducted a post-hoc sub-group analysis to examine area under ROC curve stratified by *Pseudomonas* infection status (positive vs. negative) and median baseline lung function (below median FEV1% predicted vs. equal to or above median FEV1% predicted).

Lastly, biomarker levels when a patient was clinically stable vs. experiencing a pulmonary exacerbation were compared using a paired t-test, following log transformation to normalize the data if the paired differences were not normally distributed. P-values of less than 0.05 were considered statistically significant (two-tailed test). All analyses were conducted using STATA 12.1 (College Station, TX) and figures were created using GraphPad PRISM 6 (LaJolla, CA).

## Results

### Patient Demographics

The study population was generally representative of patients typically followed at an adult CF clinic with a few notable exceptions (**Table 1**). There was a slight over-representation of males and the prevalence of CF-related diabetes was lower than reported in the literature, as it was stringently defined based on the need for insulin therapy. Seven of 30 (23%) patients were diagnosed with an exacerbation requiring IV antibiotics in the year prior to enrolment.

**Table 1:** Baseline patient characteristics.

Baseline Characteristic*	All Participants(N = 30)	Participants with paired stable-exacerbation visits (N = 15)	Participants with three consecutive stablevisits (N = 10)
**Age (years)**	28 (20–37)	27 (21–33)	27 (22–34)
**Male**	19 (63%)	11 (73%)	5 (50%)
**FEV1% predicted**	68 (39–96)	66 (35–92)	78 (59–92)
**BMI (kg/m^2^)**	22 (20–26)	22 (21–23)	23 (22–24)
***Pseudomonas*** ** culture (+)**	16 (53%)	10 (67%)	3 (30%)
**CF-related diabetes (on insulin)**	5 (17%)	1 (7%)	2 (20%)
**Use of IV antibiotics in the prior year**	7 (23%)	5 (33%)	2 (20%)
**Number of exacerbations requiring IV antibiotics in the prior year**	0 (0–0)	0 (0–1)	0 (0–0)

*Median (interquartile range) or Number (proportion).

Abbreviations: BMI = body mass index; FEV1 = forced expiratory volume in 1 second; IV = intravenous.

### Blood Biomarker Levels and Stable Lung Function

CRP and G-CSF levels inversely correlated with FEV1% predicted at the time of initial assessment, when the patients were deemed clinically stable (**Table 2**). There was also an inverse relationship between MPO level and FEV1% predicted, but did not reach statistical significance (p = 0.09). sCD14, IL-6, IL-1β, MCP-1, and VEGF levels were not related with lung function measurements during clinical stability.

**Table 2:** Correlation between blood biomarker levels and stable (baseline) lung function.

Biomarker	N	Rho (ρ)	P-value†
**sCD14**	30	−0.15	0.4
**CRP**	30	−0.42	0.02
**IL-6**	29*	0.16	0.4
**IL-1β**	23*	−0.27	0.2
**MCP-1**	30	0.11	0.6
**G-CSF**	30	−0.49	0.01
**MPO**	30	−0.31	0.09
**VEGF**	26*	0.26	0.2

*Missing individuals had undetectable levels.

† Spearman’s rank correlation.

Abbreviations: sCD14 = soluble cluster of differentiation 14; CRP = C-reactive protein; IL = interleukin; MCP-1 = monocyte chemotattractant protein-1; G-CSF = granulocyte colony-stimulating factor; MPO = myeloperoxidase; VEGF = vascular endothelial growth factor.

### Stable Visit Variability in Blood Biomarker Levels

Based on three consecutive stable visit measurements from 10 patients collected longitudinally over a median period of 9.2 (7.1 to 12.4) months, there was wide variability in levels (based on coefficient of variation) of most biomarkers except FEV1% predicted and sCD14 (**Table 3**).

**Table 3:** Coefficient of variation for blood biomarker levels over 3 consecutive stable visits.

Biomarker	N	Coefficient of Variation†
**FEV1 % Predicted**	10	4
**sCD14**	10	7
**CRP**	10	44
**IL-6**	7*	43
**IL-1β**	4*	55
**MCP-1**	10	25
**G-CSF**	6*	27
**MPO**	10	26
**VEGF**	10	52

*Missing individuals had less than 3 measurements due to undetectable levels

† Standard deviation divided by mean

Abbreviations: sCD14 = soluble cluster of differentiation 14; CRP = C-reactive protein; IL = interleukin; MCP-1 = monocyte chemoattractant protein-1; G-CSF = granulocyte colony-stimulating factor; MPO = myeloperoxidase; VEGF = vascular endothelial growth factor

### Stable Visit Blood Biomarker Levels in Patients who did vs. did not have an Exacerbation Requiring IV Antibiotics within 4 Months

**Figure 2:**
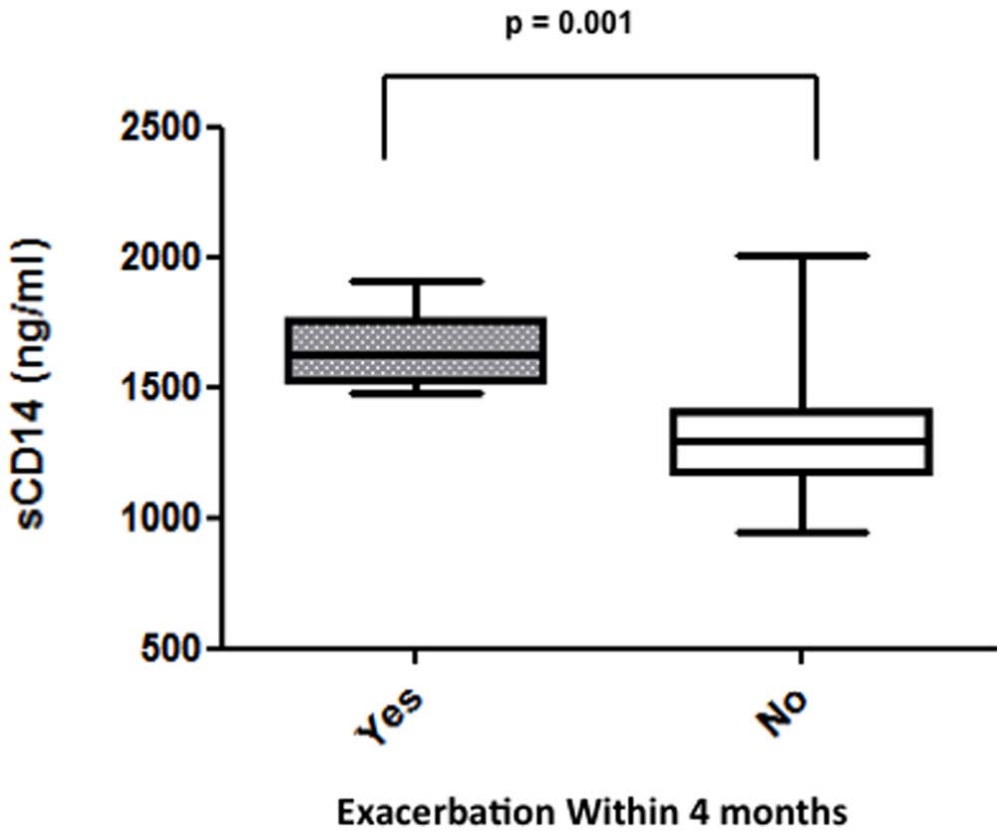
Boxplot of sCD14 levels in patients who did vs. did not experience a pulmonary exacerbation within 4 months.

Eight out of 30 (27%) patients were diagnosed with a pulmonary exacerbation requiring IV antibiotics within 4 months of the stable visit. sCD14 levels measured at the time of stable assessment were significantly higher in patients who went on to develop a pulmonary exacerbation in 4 months, compared to those who did not (p = 0.001) (Figure 2). No other biomarker levels were significantly different in patients who did vs. did not exacerbate within 4 months of a stable blood draw (Table 4).

**Table 4:** Comparison of blood biomarker levels in patients who did vs. did not experience a pulmonary exacerbation requiring IV antibiotics within 4 months of a stable blood collection.

	Exacerbation within 4 months*		
Biomarker	Yes (n = 8)	No (n = 22)	P-value†
**sCD14 (ng/mL)**	1645.7 (1.1)	1297.4 (1.2)	0.001
**CRP (mg/L)**	4.1 (5.3)	2.1 (3.5)	0.2
**IL-6 (pg/mL)**	2.1 (2.2)	2.8 (3.4)	0.5
**IL-1β (pg/mL)**	0.5 (2.0)	0.5 (1.8)	0.9
**MCP-1 (pg/mL)**	95.4 (1.2)	102.3 (1.5)	0.7
**G-CSF (pg/mL)**	53.0 (3.4)	22.1 (2.8)	0.1
**MPO (ng/mL)**	454.8 (1.6)	319.6 (1.8)	0.1
**VEGF (pg/mL)**	53.0 (3.4)	22.1 (2.3)	0.8

*Geometric mean (standard deviation)

† Two-group t-test

Abbreviations: sCD14 = soluble cluster of differentiation 14; CRP = C-reactive protein; IL = interleukin; MCP-1 = monocyte chemoattractant protein-1; G-CSF = granulocyte colony-stimulating factor; MPO = myeloperoxidase; VEGF = vascular endothelial growth factor

### sCD14 Level and the Short-term Risk of Exacerbation Requiring IV Antibiotics

**Figure 3:**
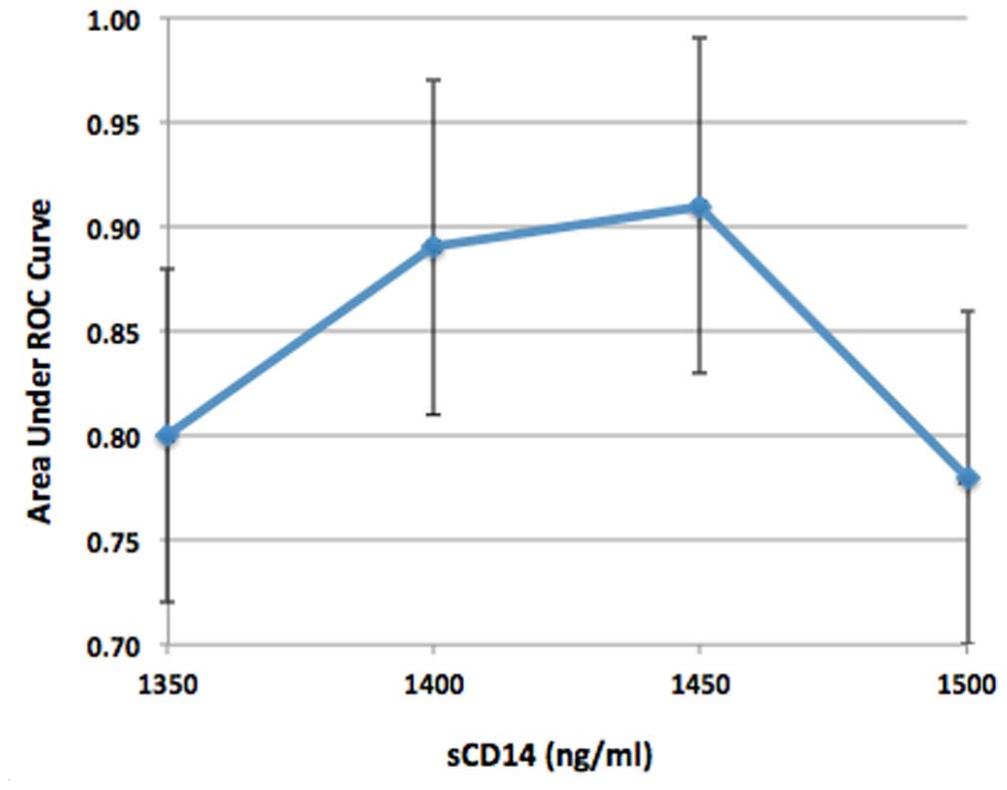
Defining an optimal sCD14 threshold value to predict pulmonary exacerbations requiring IV antibiotics within 4 months.

Various sCD14 cut-off values were examined and a cut-off value of 1450 ng/mL resulted in a maximum area under the ROC curve of 0.91 (95% CI 0.83–0.99) (**Figure 3**). This cut-off corresponded to a sensitivity of 100% (8 of 8 patients) and specificity of 82% (18 of 22 patients). In a post-hoc sub-group analysis comparing ROC curves according to baseline *Pseudomonas* infection status (positive vs. negative) and lung disease severity (below vs. equal to or above median value of 75% predicted), the area under ROC curves were similar across all sub-groups (data not presented).

### Paired Stable and Exacerbation Biomarker Measurements

Fifteen out of 30 (50%) patients had paired stable-exacerbation blood samples collected during consecutive CF clinic visits. The median time between paired samples was 3.0 (2.1 to 4.9) months. There was a significant decrease in the geometric mean FEV1% predicted from stable to exacerbation measurement (**Table 5**). Six out of fifteen (40%) exacerbations required treatment with IV antibiotics and the remainder were treated with oral antibiotics. Six out of fifteen (40%) patients had a new bacterium isolated from their exacerbation sputum culture relative to their stable sputum culture (3 *Haemophilus influenza*, 1 *Burkholderia cepacia complex* – genomovar multivorans, 1 *Streptococcus pneumonia*, and 1 *Acinetobacter baumanni*). sCD14 was the only biomarker that changed significantly from paired stable to exacerbation measurements (p = 0.03) (**Table 5**). There was a trend toward a significant increase of CRP, MCP-1, and MPO levels from stable to exacerbation measurements (all p<0.10). Subgroup analyses based on potential predictors of more severe systemic inflammation at the time of exacerbation, such as the need for IV antibiotics and growth of new bacteria on sputum culture, did not significantly modify any of the results (data not presented).

**Table 5:** Comparison of paired stable-exacerbation biomarker levels.

Biomarker	N	Stable*	Exacerbation*	P-value†
**FEV1 % Predicted**	15	64.3 (1.4)	54.6 (1.5)	0.0002
**sCD14 (ng/mL)**	15	1357.3 (1.2)	1475.0 (1.2)	0.03
**CRP (mg/L)**	15	4.1 (3.6)	6.7 (3.0)	0.09
**IL-6 (pg/mL)**	14**	2.2 (2.6)	3.7 (2.3)	0.2
**IL-1β (pg/mL)**	9**	0.7 (1.8)	0.9 (1.6)	0.8
**MCP-1 (pg/mL)**	15	95.8 (1.2)	111.7 (1.4)	0.06
**G-CSF (pg/mL)**	15	46.1 (2.9)	58.4 (3.1)	0.4
**MPO (ng/mL)**	15	383.2 (1.9)	478.9 (1.8)	0.08
**VEGF (pg/mL)**	15	10.2 (1.8)	10.0 (1.6)	0.8

*Geometric mean (standard deviation)

**Missing individuals had undetectable levels

† Paired t-test

Abbreviations: sCD14 = soluble cluster of differentiation 14; CRP = C-reactive protein; IL = interleukin; MCP-1 = monocyte chemoattractant protein-1; G-CSF = granulocyte colony-stimulating factor; MPO = myeloperoxidase; VEGF = vascular endothelial growth factor

## Discussion

New biomarkers of CF lung disease activity are urgently required to enhance clinical decision-making and improve exacerbation outcomes. Given the adverse impact that exacerbation events have on CF patients, it is a priority to prevent them. A biomarker to identify individuals that are at elevated short-term risk of developing an exacerbation will allow for more selective targeting of preventive therapies (such as mucolytics and inhaled antibiotics) in those most likely to benefit. Discovery of a biomarker that reliably increases early in the course of an exacerbation is also desirable as it may lead to earlier detection and treatment, prior to the establishment of irreversible lung damage.

In this study, we examined several biologically plausible biomarkers of disease activity that could be measured in the systemic circulation. Blood is an attractive biological compartment for biomarker measurement in CF as it can be obtained non-invasively in most clinical laboratories and has the potential for clinical application in patients of all ages and with all stages of lung disease severity. First, we examined whether stable visit biomarker levels were different in patients who were prone to experiencing an exacerbation requiring IV antibiotics within 4 months, compared to those who did not. sCD14 levels were significantly higher in patients who experienced an exacerbation in short-term follow-up. In identifying patients who do vs. do not exacerbate within 4 months of a stable clinic visit, test performance accuracy was optimized using a sCD14 cut-off value of 1450 ng/mL, with an area under ROC curve of 0.91 (95% CI 0.83–0.99). sCD14 possesses other desirable characteristics as a disease activity biomarker as it does not relate to underlying lung disease severity (i.e. FEV1% predicted) and exhibits minimal measurement variability over time when a patient is deemed clinically stable. sCD14 is biologically plausible as a biomarker of disease activity as it is involved in the innate immune response to infection and can mediate phagocytosis by binding to bacterial ligands, such as lipopolyassacharide (LPS), on the surface of gram-negative bacteria [11], [12]. It is secreted in a protease-independent manner by monocytes and is cleaved from membrane-bound forms of CD14 on the surface of monocytes and macrophages by proteases such as neutrophil elastase [13]. It is also released as class 2 acute phase proteins from the liver (in response to IL-6), possibly bridging the gap between innate and adaptive immunity [14]. sCD14 has showed promise as a biomarker of pneumonia in children and sepsis in adults [13], [15], [16].

**Figure 4:**
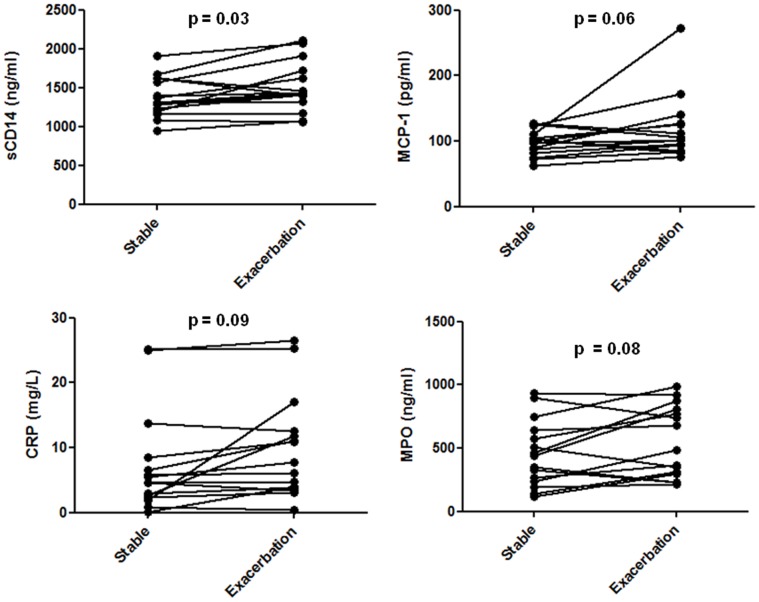
Paired stable-exacerbation biomarker levels for biomarkers with statistically significant (<0.05) or borderline significant change (p<0.10).

In our comparison of biomarker levels when a patient was clinically stable vs. experiencing a pulmonary exacerbation, the only biomarker (other than FEV1% predicted) that demonstrated significant change in paired analysis was sCD14. There was also a trend toward a significant increase in CRP, MCP-1, and MPO. For each biomarker, there was substantial overlap observed in stable and exacerbation biomarker levels between patients, which makes the establishment of an absolute cut-off for normal vs. abnormal highly unlikely, which may limit clinical application (**Figure 3**). Moreover, while most patients had an increase in sCD14, CRP, MCP-1 and MPO levels when transitioning from a stable to exacerbation health state, some individual patients had no change or even a slight decrease in biomarker levels (**Figure 4**).

There are several potential limitations to our study. First, this was a pilot study so we selected all 15 patients with paired stable-exacerbation visits and an additional 15 patients of those remaining at random. This aspect of our study design potentially limits the generalizability of our findings but unlikely introduced any systematic bias as this was defined *a priori*. For prediction of exacerbations within 4 months, such events were defined based on IV antibiotic requirement, which allowed for more reliable identification of these events through chart review, as exacerbations requiring oral antibiotics are inconsistently documented in our clinic and are confounded by the fact that almost all patients in the clinic are prophylactically treated with oral macrolides (or a similar antibiotic for those intolerant to macrolides) for exacerbation prevention. This definition resulted in the selection of more severe exacerbations with the likely omission of milder events. Another potential limitation of this definition is that the decision to treat an exacerbation with IV (vs. oral) antibiotics may extend beyond severity of the attack and may relate to the discretion of the treating physician or the preference of the patient. Notwithstanding, it is clinically relevant to focus on the prediction of exacerbations requiring IV antibiotics as they have the greatest impact on patient quality of life and survival and thus should be prioritized for prevention [17], [18].

In contrast to the prediction analysis, which exclusively relied on chart review for determining exacerbations, we prospectively collected exacerbation information for the paired analysis. Thus, we could reliably ascertain both mild exacerbations, requiring only oral antibiotics as well as more severe exacerbations requiring IV antibiotic therapy. Indeed, most of the exacerbations determined prospectively were mild events. The enrichment of mild events in the paired analysis may explain the lack of significant change observed in CRP and MPO levels from stable to exacerbation state, both of which have been shown to increase during exacerbations in prior studies [8], [19], [20], [21], [22], [23]. We chose four months as the exacerbation prediction interval of interest as most patients are followed-up routinely in the CF clinic every 3 to 4 months and therefore prediction of an event prior to the next clinic visit has the most clinical utility. In a sensitivity analysis, we did not observe a significant difference in the prediction accuracy of sCD14 if the prediction interval was changed to 3 or 6 months (data not presented). Due to our small sample size, we were limited in our ability to combine clinical variables and biomarkers into a multivariate prediction model. However, the high performance accuracy observed for sCD14 alone makes it unlikely that clinical variables would have further improved prognostic accuracy. The small sample size also precluded our ability to identify a high-risk subgroup that is more likely to benefit from blood biomarker analysis.

In conclusion, plasma sCD14 holds promise as a biomarker to identify individuals at elevated short-term risk of a pulmonary exacerbation requiring IV antibiotics but these results must be confirmed in a larger, independent cohort. No plasma biomarkers demonstrated sufficient discriminatory power for the detection of pulmonary exacerbations and therefore new biomarkers of disease activity must be discovered to enhance the early detection of exacerbations.
